# Estrogen Signaling Inhibits the Expression of *anti-Müllerian hormone* (*amh*) and *gonadal-soma-derived factor* (*gsdf*) during the Critical Time of Sexual Fate Determination in Zebrafish

**DOI:** 10.3390/ijms25031740

**Published:** 2024-02-01

**Authors:** Yonglin Ruan, Xuehui Li, Gang Zhai, Qiyong Lou, Xia Jin, Jiangyan He, Zhan Yin

**Affiliations:** 1 State Key Laboratory of Freshwater Ecology and Biotechnology, Institute of Hydrobiology, Chinese Academy of Sciences, Wuhan 430072, China; ruanyl@ihb.ac.cn (Y.R.); lixuehui22@mails.ucas.ac.cn (X.L.); louqiyong@ihb.ac.cn (Q.L.); jyhe@ihb.ac.cn (J.H.); 2 College of Advanced Agricultural Sciences, University of Chinese Academy of Sciences, Beijing 100049, China; 3 Hubei Hongshan Laboratory, Huazhong Agriculture University, Wuhan 430070, China; 4 The Innovative Academy of Seed Design, Chinese Academy of Sciences, Wuhan 430072, China

**Keywords:** estrogen signaling, *amh*, *gsdf*, gonad, sex differentiation

## Abstract

The mechanism of fish gonadal sex differentiation is complex and regulated by multiple factors. It has been widely known that proper steroidogenesis in Leydig cells and sex-related genes in Sertoli cells play important roles in gonadal sex differentiation. In teleosts, the precise interaction of these signals during the sexual fate determination remains elusive, especially their effect on the bi-potential gonad during the critical stage of sexual fate determination. Recently, all-testis phenotypes have been observed in the *cyp17a1*-deficient zebrafish and common carp, as well as in *cyp19a1a*-deficient zebrafish. By mating *cyp17a1*-deficient fish with transgenic zebrafish *Tg(piwil1:EGFP-nanos3UTR)*, germ cells in the gonads were labelled with enhanced green fluorescent protein (EGFP). We classified the *cyp17a1*-deficient zebrafish and their control siblings into primordial germ cell (PGC)-rich and -less groups according to the fluorescence area of the EGFP labelling. Intriguingly, the EGFP-labelled bi-potential gonads in *cyp17a1*+/+ fish from the PGC-rich group were significantly larger than those of the *cyp17a1*−/− fish at 23 days post-fertilization (dpf). Based on the transcriptome analysis, we observed that the *cyp17a1*-deficient fish of the PGC-rich group displayed a significantly upregulated expression of *amh* and *gsdf* compared to that of control fish. Likewise, the upregulated expressions of *amh* and *gsdf* were observed in *cyp19a1a*-deficient fish as examined at 23 dpf. This upregulation of *amh* and *gsdf* could be repressed by treatment with an exogenous supplement of estradiol. Moreover, tamoxifen, an effective antagonist of both estrogen receptor α and β (ERα and Erβ), upregulates the expression of *amh* and *gsdf* in wild-type (WT) fish. Using the *cyp17a1*- and *cyp19a1a*-deficient zebrafish, we provide evidence to show that the upregulated expression of *amh* and *gsdf* due to the compromised estrogen signaling probably determines their sexual fate towards testis differentiation. Collectively, our data suggest that estrogen signaling inhibits the expression of *amh* and *gsdf* during the critical time of sexual fate determination, which may broaden the scope of sex steroid hormones in regulating gonadal sex differentiation in fish.

## 1. Introduction

The regulation of gonadal sex differentiation is complex and regulated by multiple factors. In vertebrates, genetic sex determination (GSD) has been classified into monogenic systems and polygenic systems to determine their entry into male or female differentiation pathways [1,2]. For polygenic systems, in zebrafish (*Danio rerio*), Malawi cichlid fish (*Metriaclima zebra*), and European sea bass (*Dicentrarchus labrax*), sex is determined by allelic combinations of several loci dispersed throughout the genome or located on a preferential pair of (sex) chromosomes [3,4,5,6,7]. Studying the regulatory function of genetic factors in zebrafish, which may be conserved in all vertebrates for gonadal differentiation, will help to decipher the mechanisms that determine sexual fate.

Natural sex hormones, including androgen and estrogen, which are synthetized in the gonad Leydig cells, exert a wide range of biological effects on the body, affecting the growth and function of the reproductive organs and the development of secondary sexual characteristics [8]. In zebrafish, domesticated experimental strains have lost their natural sex determinants, and they lack a single strong sex genetic determinant [9]. However, the sex ratio of zebrafish exhibits significant plasticity under exogenous sex hormone or aromatase inhibitor treatment. Zebrafish treated with estradiol from the juvenile stage result in a female-biased population [10], whereas long-term use of aromatase inhibitors causes an absolute sex reversal from female to phenotypic male in zebrafish [11]. Moreover, a series of zebrafish mutation models also elucidate the important function of sex hormones in sex differentiation. *Cyp17a1*−/− and *cyp17a1*−/−;*androgen receptor (ar)* −/−zebrafish all exhibit the all-testis phenotype [12], indicating that the androgen signaling is dispensable for testicular differentiation, while estrogen derived from androgen is essential for ovarian differentiation. On the other hand, *cyp19a1a*-deficient zebrafish mutants generated from different laboratories were reported to be the phenotypical all-male fish [13,14,15]. Although the important role of estrogen in sex differentiation has been extensively investigated, the underlying mechanism of the all-male phenotype caused by *cyp17a1*- or *cyp19a1a*-deficiency is still unascertained.

The function of numerous Sertoli cell-related genes have been elucidated, including *ar*, *dmrt1*, *amh*, *gsdf*, etc. The *ar* mutant zebrafish exhibited defective spermatogenesis and significant skew in the sex ratio towards females (nearly 62%) [16]. In another study with male zebrafish lacking *ar*, defective steroidogenesis in Leydig cells and disorganized testicular development were observed [17]. Moreover, *dmrt1* was found to be expressed in both Sertoli cells and the germ line of the testes, and its deficiency led to defective testis development and a female-biased sex ratio [18,19]. *Amh*, a member of the *Tgfb* superfamily, was reported to express in Sertoli cells and zebrafish males. *Amh*-deficient zebrafish displayed a female biased sex ratio (nearly 71%) [20]. It is also non-negligible that the up-regulated expression of *amh* was seen in the *fancl* mutant zebrafish, which all developed into males at the critical stage of sex differentiation [21]. In contrast, in other studies, the *amh* mutant did not exhibit much difference in the sex ratio compared to the control siblings [22,23]. These paradoxes hamper the understanding of the role of *amh* in determining gonadal differentiation. Moreover, *gsdf*, another member of the *Tgfb* superfamily, regulates ovarian follicle maturation and the expression of genes for steroid biosynthesis and female fertility, but it is not the primary genetic sex determinant in zebrafish [24]. Therefore, more evidence is needed to elucidate the function of the sex-related genes, especially via characterizing the precise interaction of the signals derived from the Leydig cells and Sertoli cells during the sexual fate determination. 

Gonads in zebrafish initially form an ovary-like structure (termed the “bipotential juvenile ovary”), which subsequently develops either into the ovaries in females or testes in males [25,26]. The classical viewpoint is that the bi-potential gonads start to differentiate into an ovary or a testis during 20 to 25 dpf in zebrafish [27]. In the transgenic zebrafish *Tg(piwil1:EGFP-nanos3UTR)*, the germ cells are labelled with enhanced green fluorescent protein (EGFP), making it easier to identify the area of the presumptive gonads. From the stage at 20 dpf, the dimorphic sizes of presumptive gonads become obvious. Juveniles with big gonads (PGC-rich) mainly develop into females, and those with small gonads (PGC-less) mainly develop into males [28].

In this study, we monitored the primordial germ cell (PGC) dynamics in the *cyp17a1*- and *cyp19a1a*-deficient zebrafish, which facilitates the evaluation of gene expressions by transcriptome analysis at 23 dpf, the critical stage of sexual differentiation. We demonstrated the inhibitory effect of estrogen on *amh* and *gsdf* by two different estrogen-deficient zebrafish models, providing a novel insight into the regulatory role of estrogen signaling in tipping the bi-potential gonads towards ovarian differentiation of female fate.

## 2. Results

### 2.1. Estrogen Enlarged the Bi-Potential Gonad in Zebrafish

**Figure 1:**
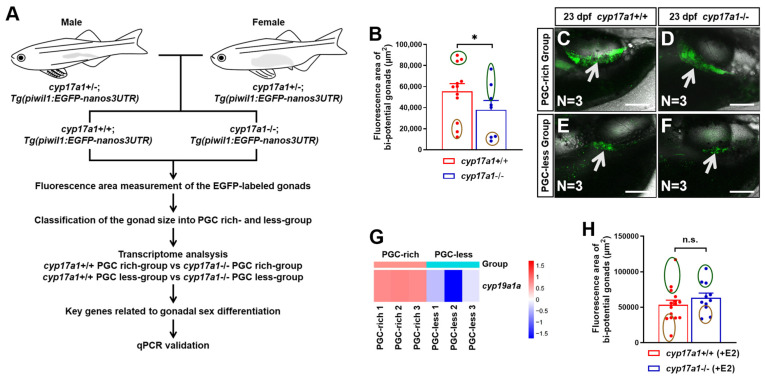
Estrogen enlarged the bi-potential gonad in zebrafish. (**A**) A schematic showing the procedure of the classification according to PGC area and gene expression analyses. (**B**) The bi-potential gonad area of the selected *cyp17a1*+/+ and *cyp17a1*−/− fish at 23 dpf. Dark green circles: top three bipotential gonad area. Brown circles: last three bi-potential gonad area. (**C**,**D**) Representative images of the selected *cyp17a1*+/+ and *cyp17a1*−/− fish from the PGC-rich group at 23 dpf. EGFP-labeled gonads were pointed by white arrows. Scale bar, 250 µm. (**E**,**F**) Representative images of the selected *cyp17a1*+/+ and *cyp17a1*−/− fish from the PGC-less group at 23 dpf. EGFP-labeled gonads were pointed by white arrows. Scale bar, 250 µm. (**G**) Gene expression heat map of *cyp19a1a* in *cyp17a1*+/+ zebrafish from PGC-rich and PGC-less groups at 23 dpf. (**H**) The bi-potential gonad area of the selected *cyp17a1*+/+ and *cyp17a1*−/− fish with estradiol treatment from 16 to 23 dpf. E2, 17 β-estradiol. n.s., no significance. * *p* < 0.05.

To monitor the bi-potential gonad development, the mating between *cyp17a1*−+/− fish and *Tg(piwil1:EGFP-nanos3UTR)* fish were performed. Subsequently, the *cyp17a1*+/−;*Tg(piwil1:EGFP-nanos3UTR)* fish of the F1 population were crossed to obtain the F2 population, among which, the *cyp17a1*−/− fish and *cyp17a1*+/+ fish of their control siblings (control fish) were used for the fluorescence area measurement, as well as transcriptome and qPCR analysis (Figure 1A). The fluorescence area of the EGFP-labeled gonads in *cyp17a1*-deficient fish was smaller than that of the control fish from PGC-rich and PGC-less groups (N = 12 and 8 in control fish and *cyp17a1*-deficient fish, respectively) (Figure 1B). The control fish and *cyp17a1*-deficient fish with the top and last three bi-potential gonad area were termed as PGC-rich and PGC-less groups, respectively, and were selected for sampling for the transcriptome and qPCR analyses in the present study (Figure 1C–F). In the comparative transcriptome between *cyp17a1*+/+ fish of the PGC-rich group and PGC-less group, the upregulated expression of *cyp19a1a* was observed in the gonadal samples of *cyp17a1*+/+ fish of the PGC-rich group (*p* = 0.028822) (Figure 1G). Moreover, when *cyp17a1*-deficient fish were treated with 0.1 μg/L exogenous estradiol from 16 to 23 dpf, their fluorescence area of the EGFP-labeled gonads became comparable to those of estradiol-treated control siblings (N=15 and 11 in control fish and *cyp17a1*-deficient fish, respectively) (Figure 1H). Considering the impaired estrogen synthesis of the *cyp17a1*-deficient fish, our results suggest that estrogen is positively correlated with the development of bi-potential gonads during the critical time of sexual fate determination in zebrafish. 

### 2.2. Upregulated Expression of amh and gsdf Was Observed in cyp17a1−/− Fish of the PGC-Rich Group

**Figure 2:**
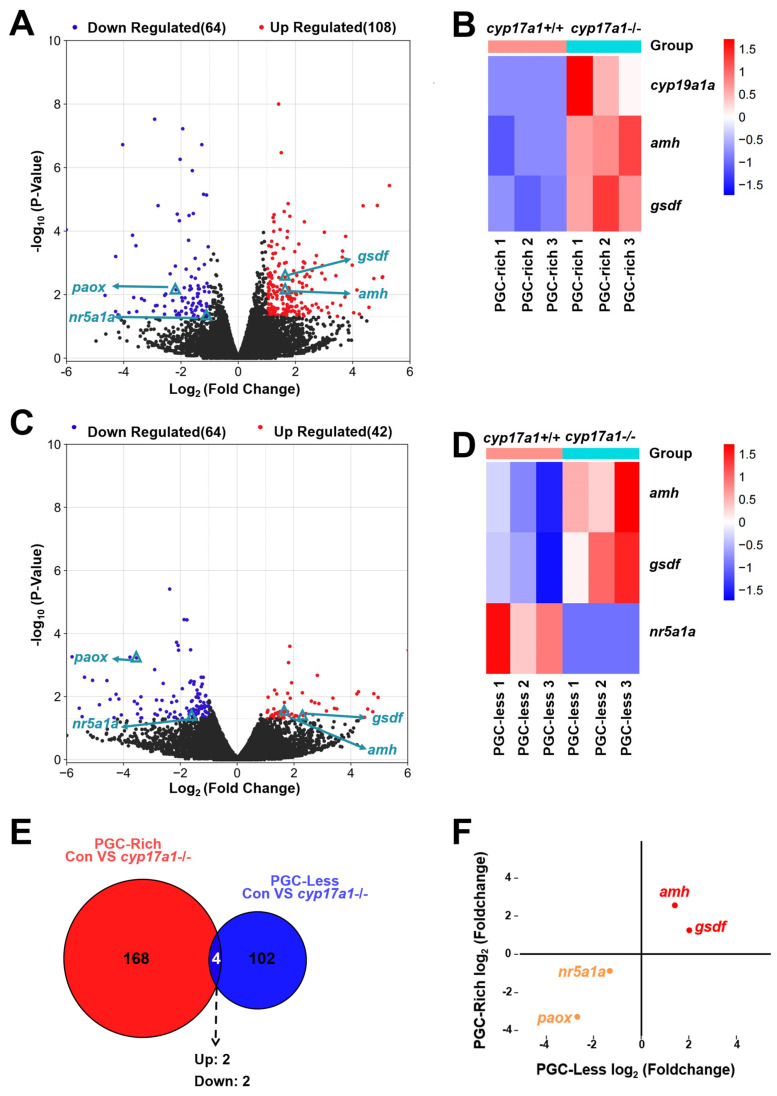
Gene expression heat map and gene set enrichment analysis. (**A**) DEGs from the comparison between *cyp17a1*+/+ and *cyp17a1*−/− fish of the PGC-rich group at 23 dpf. Volcano plot showing genes, including *amh* and *gsdf*, that were differentially expressed in *cyp17a1*+/+ and *cyp17a1*−/− fish of the PGC-rich group at 23 dpf. (**B**) Gene expression heat map of gonadal differentiation of *cyp17a1*+/+ and *cyp17a1*−/− fish of the PGC-rich group at 23 dpf. (**C**) DEGs from the comparison between *cyp17a1*+/+ and *cyp17a1*−/− fish of the PGC-less group at 23 dpf. Volcano plot showing genes, including *amh* and *gsdf*, that were differentially expressed in *cyp17a1*+/+ and *cyp17a1*−/− fish of the PGC-less group at 23 dpf. (**D**) Gene expression heat map of gonadal differentiation of *cyp17a1*+/+ and *cyp17a1*−/− fish of the PGC-less group at 23 dpf. (**E**) Veen analysis of DEGs. Up: upregulated. Down: downregulated. (**F**) Scatter plot of the intersected DEGs. Red dots: co-upregulated. Orange dots: co-downregulated.

Subsequently, to identify the genes most likely to be altered by *cyp17a1*-depletion and thereafter estrogen synthesis impairment, the transcriptome analysis of the *cyp17a1*+/+ fish and *cyp17a1*−/− fish from the PGC-rich group was conducted. In the comparison between the PGC-rich gonadal samples from cyp17a1+/+ fish and cyp17a1−/− fish, a total of 172 genes were differentially expressed as statistically analyzed with FPKM (fragments per kilobase of exon model per million mapped fragments) (Table S1). Compared to *cyp17a1*+/+ fish of the PGC-rich group, 108 genes were upregulated and 64 genes were downregulated in *cyp17a1*−/− fish (Figure 2A). Compared to that of the *cyp17a1*+/+ fish from the PGC-rich group, the ovarian-differentiation-related gene *cyp19a1a* and testis-differentiation-related genes *amh* and *gsdf* were upregulated in the *cyp17a1*−/− fish (Figure 2B).

In the comparison between the PGC-less gonadal samples from *cyp17a1*+/+ fish and *cyp17a1*−/− fish, a total of 106 genes were differentially expressed (Table S2). Compared to *cyp17a1*+/+ fish of PGC-less, 42 genes were upregulated and 64 genes were downregulated in *cyp17a1*−/− fish (Figure 2C). Unlike the upregulated genes *amh* and *gsdf*, which are testis differentiation related, in *cyp17a1*−/− fish, the expression of *nuclear receptor subfamily 5, group A, member 1a* (*nr5a1a*), which was known necessary for the maintenance of oocytes, was downregulated (Figure 2D). 

To characterize the intersected genes that were differentially expressed in *cyp17a1*−/− fish of PGC-rich and PGC-less groups, the 172 and 106 DEGs from the comparison of *cyp17a1*+/+ fish and *cyp17a1*−/− fish from PGC-rich and PGC-less groups were analyzed integratively. Based on the intersected result of the Veen analysis, only four genes were identified as intersected, namely, *amh*, *gsdf*, *nr5a1a*, and *polyamine oxidase* (*paox*) (Figure 2E). Among the four intersected DEGs, *amh*/*gsdf* and *nr5a1a*/*paox* were up- and downregulated in *cyp17a1*−/− fish of PGC-rich and PGC-less groups, respectively (Figure 2F).

### 2.3. Estrogen Treatment Downregulated the Expression of amh and gsdf in cyp17a1−/− Zebrafish Gonadal Tissues

**Figure 3:**
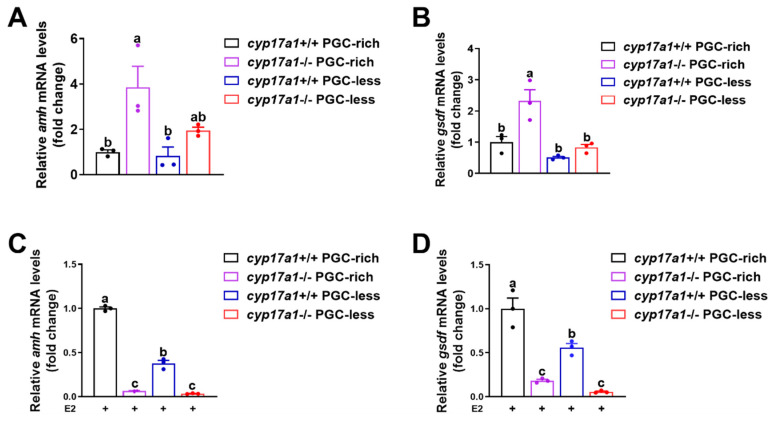
The transcriptome results could be validated with qPCR, but downregulated *amh* and *gsdf* were observed in *cyp17a1*−/− fish after estradiol treatment from 16 to 23 dpf. (**A**,**B**) Relative expression of *amh and gsdf* in *cyp17a1*+/+ and *cyp17a1*−/− fish of PGC-rich and PGC-less groups at 23 dpf with qPCR. Upregulated expression of *amh* and *gsdf* was observed in *cyp17a1*−/− fish of PGC-rich and PGC-less groups at 23 dpf. (**C**,**D**) Relative expression of *amh and gsdf* in *cyp17a1*+/+ and *cyp17a1*−/− fish of PGC-rich and PGC-less groups treated with estradiol from 16 to 23 dpf. E2, 17 β-estradiol. Different letters in the bar charts represent significant differences.

To validate the transcriptomic results, another independent sampling from at 23 dpf and qPCR were performed to test the expression of *amh* and *gsdf*. The observations confirmed that *amh* and *gsdf* exhibited a consistent expression profile as observed in the RNA-seq data, e.g., *amh* and *gsdf* were upregulated in *cyp17a1*−/− fish from PGC-rich and PGC-less groups at 23 dpf (Figure 3A,B). 

To examine whether the upregulated expression of *amh* and *gsdf* in *cyp17a1*−/− zebrafish of the PGC-rich group was caused by the impaired estrogen synthesis, the exogenous estradiol was supplemented to monitor their expression after administration from 16 to 23 dpf. Significantly, compared to those of the *cyp17a1*+/+ zebrafish, the expression of *amh* and *gsdf* was significantly downregulated in *cyp17a1*−/− zebrafish of PGC-rich and PGC-less groups after estradiol treatment (Figure 3C,D). These results indicated that the expressions of *amh* and *gsdf* in *cyp17a1*−/− zebrafish at 23 dpf were negatively responsive to the exogenous estradiol supplement. 

### 2.4. Upregulated Expression of amh and gsdf Was Observed in cyp19a1a−/− Fish of the PGC-Rich Group

**Figure 4:**
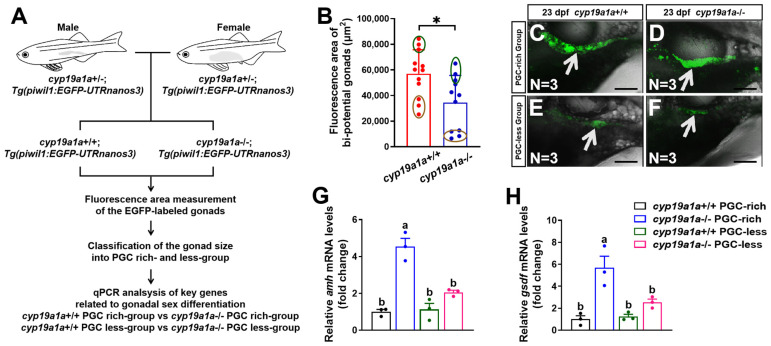
Upregulated expression of *amh* and *gsdf* was observed in *cyp19a1a*−/− fish of PGC-rich and PGC-less groups at 23 dpf. (**A**) A schematic showing the procedure of the classification according to PGC area and gene expression analyses. (**B**) The bi-potential gonad area of the selected *cyp19a1a*+/+ and *cyp19a1a*−/− fish at 23 dpf. Dark green circles: top three bipotential gonad area. Brown circles: last three bi-potential gonad area. (**C**,**D**) Representative images of the selected *cyp19a1a*+/+ and *cyp19a1a*−/− fish from the PGC-rich group at 23 dpf. EGFP-labeled gonads were pointed by white arrows. Scale bar, 300 µm. (**E**,**F**) Representative images of the selected *cyp19a1a*+/+ and *cyp19a1a*−/− fish from the PGC-less group at 23 dpf. EGFP-labeled gonads were pointed by white arrows. Scale bar, 300 µm. (**G**,**H**) Relative expression of *amh and gsdf* in *cyp19a1a*+/+ and *cyp19a1a*−/− fish of the PGC-rich and PGC-less groups at 23 dpf. n.s., no significance. * *p* < 0.05. Different letters in the bar charts represent significant differences.

Besides *cyp17a1*-deficient zebrafish, *cyp19a1a*-deficient zebrafish is another excellent model to investigate the effect of estrogen on the expression of the sex-related genes caused by estrogen synthesis insufficiency. To verify our hypothesis that estrogen inhibits the expression of *amh* and *gsdf* during the critical time of sexual fate determination in zebrafish, we examined the expression profiles of *amh* and *gsdf* in *cyp19a1a*−/− zebrafish. The F2 population from the inbreeding of *cyp19a1a*+/−;*Tg(piwil1:EGFP-nanos3UTR)* females and males of the F1 population were sampled at 23 dpf (Figure 4A). Similarly, the fluorescence area of the EGFP-labeled gonads of *cyp19a1a*-deficient fish was smaller than those of *cyp19a1a*+/+ fish of their control siblings (control fish) from the PGC-rich and PGC-less groups (N = 12 and 11 in control fish and *cyp19a1a*-deficient fish, respectively) (Figure 4B). The *cyp19a1a*-deficient fish and their control siblings with the top and last three bi-potential gonad area were termed as PGC-rich and PGC-less groups, respectively, and were selected for sampling for the gene expression analyses (Figure 4C–F). The expression of *amh* and *gsdf* was significantly upregulated in *cyp19a1a*−/− fish of the PGC-rich group (Figure 4G,H). Thus, utilizing another zebrafish model of estrogen synthesis insufficiency, we confirmed that estrogen may indeed inhibit the expression of *amh* and *gsdf* during the critical stage of sex differentiation in zebrafish.

### 2.5. Estradiol Inhibited the Expression of amh and gsdf in Zebrafish via Estrogen Receptor

**Figure 5:**
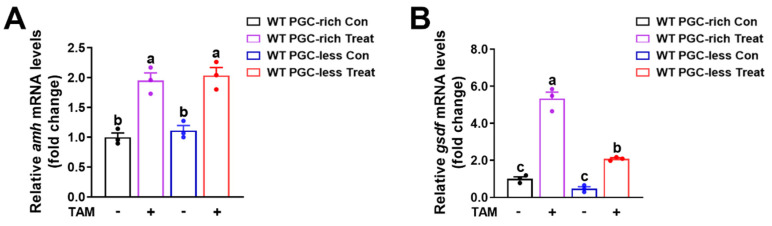
Upregulated expression of *amh* and *gsdf* was observed in WT fish of PGC-rich and PGC-less groups treated with tamoxifen from 16 to 23 dpf. (**A**) Upregulated expression of *amh* was observed in WT fish of PGC-rich and PGC-less groups treated with tamoxifen from 16 to 23 dpf. (**B**) Upregulated expression of *gsdf* was observed in WT fish of PGC-rich and PGC-less groups treated with tamoxifen from 16 to 23 dpf. Con, control. Treat, treated with TAM. TAM, tamoxifen. Different letters in the bar charts represent significant differences.

Tamoxifen is a competitive antagonist of estrogens [29,30,31]. Here, tamoxifen was used to block the endogenous estrogen signaling via competitive combination to the estrogen receptor in zebrafish. The fish of the *Tg(piwil1:EGFP-nanos3UTR)* strain were administrated 500 ng/L tamoxifen from 16 to 23 dpf. The siblings reared in the system water were used as a control. As examined in fish at 23 dpf, the significantly upregulated expressions of *amh* and *gsdf* after fish were administrated tamoxifen were observed in WT fish of PGC-rich and PGC-less groups (Figure 5A,B). These results not only confirmed that the expression of *amh* and *gsdf* in fish from PGC-rich and PGC-less groups was negatively correlated with the exogenous estrogen but also suggested that estrogen inhibits the expression of *amh* and *gsdf* via estrogen receptor in zebrafish at 23 dpf.

## 3. Discussion

In recent studies, the all-testis phenotypes have been reported in *cyp17a1*- and *cyp19a1a*-deficient zebrafish, which exhibited impaired estrogen synthesis in common with both mutated models [12,15]. In this study, by classifying the *cyp17a1*- and *cyp19a1a*-deficient fish at 23 dpf into PGC-rich and PGC-less groups and comparing them to their control siblings, the promotional function of estrogen signaling on bi-potential gonad development in zebrafish during their juvenile stage was found. Moreover, the associative signaling interaction between Leydig cells and Sertoli cells was revealed, and the inhibitory effect of estrogen signaling on Sertoli cell-related genes was identified. To our knowledge, this is the first study of the gonadal sex differentiation in zebrafish that combines the depletion of the genes involved in gonadal steroidogenesis and the EGFP-labelled bi-potential gonads, and which facilitates the sampling of the fish from PGC-rich and PGC-less groups.

It has been generally accepted that gonadal sex differentiation in zebrafish is documented to occur between nearly 20 and 40 dpf, lasting for several days [15,32], during which the ovary-like gonad in juvenile zebrafish is bi-potential and will either differentiate into ovaries in females or testes in males after a transitional intersexual phase [33]. This could be validated by the upregulated expression of *cyp19a1a* among the DEGs in the *cyp17a1*+/+ fish that were PGC-rich compared to that of the PGC-less group at 23 dpf. A sufficient number of germ cells is essential for the female differentiation in zebrafish [34,35], as the loss of germ cells in *dnd* morphants leads to an all-testis differentiation in zebrafish [34,36,37]. Recently, taking advantage of the *Tg*(*piwil1-egfp-nos3UTR*) zebrafish, Ye et al. reported that a high amount of PGCs mainly developed into females and those with a low amount of PGCs mainly developed into males in zebrafish [28]. DEGs were identified via comparing zebrafish of the PGC-rich group and PGC-less group at 25 and 30 dpf, and the differentiated biological processes were enriched, in including the biological processes related to metabolic activities in the production of energy and maternal substances, RNA degradation, and DNA repair [38]. Despite this, it is curious to know the role of estrogen signaling in mediating PGC abundance in zebrafish bi-potential juvenile gonads. First, an upregulated expression of *cyp19a1a* was observed in the gonadal samples from the PGC-rich fish compared to that from the PGC-less fish. Second, the fluorescence area of the EGFP-labeled gonads in *cyp17a1*- or *cyp19a1a*-deficient fish were smaller than the control fish of PGC-rich and PGC-less groups. More importantly, when *cyp17a1*-deficient fish were treated with exogenous estradiol from 16 to 23 dpf, the fluorescence areas of the EGFP-labeled gonads become comparable with the estradiol-treated control siblings. We uncovered that in zebrafish during the critical time of sexual fate determination (as examined at 23 dpf), estrogen is positively correlated with the development of bi-potential gonads. 

The majority fish of the PGC-rich group were proposed to develop into females [28]. Thus, the impaired signaling on ovarian differentiation in fish of the PGC-rich group would then be our focus. Estrogen plays an important role in ovarian differentiation, as estradiol treatment before and during the period of sexual differentiation resulted in a significant bias towards female sex [39]. Based on the results of transcriptome and qPCR, we confirmed that *amh* and *gsdf* were upregulated in *cyp17a1*−/− fish of PGC-rich and PGC-less groups at 23 dpf. Noteworthily, in *cyp17a1*- or *cyp19a1a*-deficient fish from the PGC-less group, this upregulation was not as significant in *cyp17a1*−/− fish of the PGC-rich group. It is also noticeable that the upregulated genes in the comparison between *cyp17a1*+/+ fish and *cyp17a1*−/− fish of the PGC-less group were less than those between *cyp17a1*+/+ fish and *cyp17a1*−/− fish of the PGC-rich group (106 vs. 172). This may be owing to the abundantly expressed *cyp19a1a* in the bi-potential gonads of control fish from the PGC-rich group. That is to say, the more comprehensive response observed in the comparison between *cyp17a1*+/+ fish and *cyp17a1*−/− fish of the PGC-rich group may be caused by the significant difference in the content of estrogens. Therefore, compared to those of the *cyp17a1*- or *cyp19a1a*-deficient fish from the PGC-rich group, the control fish from the PGC-rich group may possess a higher activity in estrogen synthesis and concentration of estrogen per se, which thereby inhibits its expression of *amh* and *gsdf*. 

The *cyp17a1*- and *cyp19a1a*-deficient zebrafish are excellent models for the landscape analysis of the antagonistic effect between estrogen signaling of Leydig cells and sex-related genes of Sertoli cells [12,15], as the EGFP-labelled PGCs make it easy for the observation and classification of PGCs in the juvenile gonads of zebrafish and sample the gonads that start to differentiate for RNA sequencing. Several Sertoli cell-related genes have been documented in maintaining testis differentiation in zebrafish, including *amh*, *gsdf*, *dmrt1*, *ar*, *WT1 transcription factor a* (*wt1a*), and *sox9a* [19]. The *dmrt1* and *ar*-mutant zebrafish exhibit a significant skew in the sex ratio towards females [16,19,40]. The additional depletion of *dmrt1* from *cyp19a1a*-deficient fish resulted in the formation of ovaries containing follicles (in *cyp19a1a*−/−;*dmrt1*−/− fish) [40], providing solid evidence supporting the existence of the associated interaction between Leydig cells and Sertoli cells during gonad differentiation. Unfortunately, the paradox and unexpected views of *amh* and *gsdf* in regulating sexual differentiation were concluded [19,20,22,23,24]. Despite this, unlike the observations in fish at 25 and 30 dpf, the upregulated expression of *amh* and *gsdf*, but not *dmrt1* and *ar*, was observed in *cyp17a1*−/− fish of the PGC-rich group at 23 dpf. The expression of most steroidogenic genes, *ar,* and *dmrt1* were enriched in presumptive males [38]. We speculate that compared to *dmrt1* and *ar*, *amh* and *gsdf* may be more responsive to the compromised estrogen signaling to initiate the male differentiation. Besides *amh* and *gsdf*, the highest upregulation of *cyp19a1a* was observed in the *cyp17a1*−/− fish of the PGC-rich group. This was probably caused by the compensatory effects due to the absence of an effective negative feedback regulation of estradiol, which was also observed in the *cyp11a2* mutant zebrafish [41].

Notably, compared to those of the control siblings, *nr5a1a* and *paox* were downregulated in the *cyp17a1*−/− fish of PGC-rich and PGC-less groups. Adult gonads of *nr5a1a* mutant zebrafish at 3 months post-fertilization (mpf) contained seminiferous tubules with a few spermatocytes, but they lacked mature spermatozoa and could not induce WT females to spawn [42]. Yan et al. documented that *nr5a1a* is required for oocytes maintenance, female development, and sperm maturation [42]. Considering the essential role of *nr5a1a* in oocyte maintenance, it is reasonable to speculate that the downregulated *nr5a1a* failed to tilt the bi-potential gonads towards ovarian differentiation of female fate in *cyp17a1*−/− fish of PGC-rich and PGC-less groups. Unfortunately, without available and efficient antibodies against fish ERs, no further proof could be provided to support whether the expression of *nr5a1a* was directly regulated by estrogen signaling or not. *PAOX* is involved in polyamine metabolism and influences the oxidative balance in cells [43]. Unexpectedly, *paox*, which was poorly studied in fish, was downregulated in the *cyp17a1*−/− fish of PGC-rich and PGC-less groups. Che et al. used bioinformatics analysis based on the microarray datasets (GSE34095) to identify the DEGs as biomarkers and therapeutic targets in degenerated discs and reported that *PAOX* was a member of the 1057 DEGs observed [43]. However, it is uncertain if *paox*-mediated polyamine metabolism is implicated in the ovarian differentiation failure in *cyp17a1*−/− fish.

The *cyp17a1*−/− zebrafish all developed into males [12]. Cyp17a1 is the key enzyme in the synthesis of testosterone, the precursor of estradiol [44]. Although the expression of *cyp17a1* was not altered in *cyp17a1*−/− zebrafish, its potential role in regulating gene expression and the formation of juvenile ovary and oocyte-like germ cells should not be neglected.

Collectively, though the roles of *amh* and *gsdf* in sexual fate determination were not concluded from the previous genetic analyses, we deciphered that there is a clear antagonism between estrogen signaling and *amh* and *gsdf* in juvenile zebrafish with bi-potential gonads. The upregulated expression of *amh* and *gsdf* may promote gonadal embarkation on the testis differentiation in *cyp17a1*- or *cyp19a1a*-deficient zebrafish of the PGC-rich group. Of course, further genetic evidence by the establishment of the *cyp17a1*−/−;*amh*−/− or *cyp17a1*−/−;*gsdf*−/− zebrafish is needed to uncover the underlying roles of *amh* and *gsdf* during gonadal differentiation.

## 4. Materials and Methods

### 4.1. Fish Stocks

The transgenic zebrafish *Tg(piwil1:EGFP-nanos3UTR)* were purchased from the China Zebrafish Resource Center, National Aquatic Biological Resource Center (CZRC/NABRC, Wuhan, China). In this transgenic zebrafish *Tg(piwil1:EGFP-nanos3UTR)*, the germline can be labeled with enhanced green fluorescent protein (EGFP). Then, the transgenic line *Tg(piwil1:EGFP-nanos3UTR)* was introduced into the mutant line to generated *cyp17a1*+/−;*Tg(piwil1:EGFP-nanos3UTR)* and *cyp19a1a*+/−;*Tg(piwil1:EGFP-nanos3UTR)* fish of the F1 population, which were incrossed to establish the offspring of the F2 population containing the homozygotes. Fish were bred and maintained according to the standard procedures described in the Zebrafish Book [45]. 

### 4.2. Collection of Samples

To classify the PGC area into the PGC-rich group and PGC-less group, the fish strains of *Tg(piwil1:EGFP-nanos3UTR)* were crossed with the *cyp17a1*+/− fish to obtain the F1 population that contained the fish with the genotype of *cyp17a1*+/− *Tg(piwil1:EGFP-nanos3UTR)*. Among the F1 population, the fish of *cyp17a1*+/−*Tg(piwil1:EGFP-nanos3UTR)* were incrossed to generate the F2 population that contained the fish with the genotype of *cyp17a1*+/+;*Tg(piwil1:EGFP-nanos3UTR)*, *cyp17a1*+/−;*Tg(piwil1:EGFP-nanos3UTR)*, and *cyp17a1*−/−;*Tg(piwil1:EGFP-nanos3UTR)*. The fish of the F2 population at 23 dpf were randomly selected for image capture using a Leica SP8 confocal microscope (Leica, Weztlar, Germany). The caudal fin of fish was cut for genotyping, and the rest of the body was immediately immersed in liquid nitrogen to protect RNA from degradation for further use. Then, the area of EGFP-labeled PGCs of each fish was measured using ImageJ software (version 1.49v), and the fish were classified two groups (PGC-rich group and PGC-less group) according to the area of EGFP-labeled PGCs. 

### 4.3. Transcriptome Analysis

At the indicated time points, total RNA from zebrafish fish was extracted using TRIzol reagent (15596-026; Invitrogen, Carlsbad, CA, USA), following the standard protocol provided by the manufacturer. A single fish was performed for transcriptome analysis, and each group (the PGC-rich group and PCR-less group) was experimented on with three biological replicates. The quality and concentration of RNA were evaluated using 1% agarose gel electrophoresis and Nanodrop (Thermo Fisher, Carlsbad, CA, USA). Subsequently, the integrity of the RNA was evaluated using the RNA Nano 6000 Assay Kit of the Bioanalyzer 2100 system (Agilent Technologies, Santa Clara, CA, USA). By the analysis with RNA concentration, OD 260/280, OD260/230, RQN/RIN, and 28S/18S, the quality assessment of the extracted RNA evaluated as A-grade were adopted for the following transcriptome analysis. RNA-seq reads were generated using the Illumina NovaSeq 6000 system. For each sample, an average of 42 million clean reads were generated. A total of 44,600 transcripts and 25432 genes were detected in the samples. High-quality mRNA reads were mapped to the *Danio rerio* genome (GRCz11) using HISAT2 (version 2.2.4, http://daehwankimlab.github.io/hisat2/) (accessed on 16 October 2023). For the transcriptome analysis, the analysis of the differential expressed genes (DEGs) was performed using the DESeq2 package (v1.30.1) with a fold change of two and a *p*-value cutoff of 0.05, and the volcano plot was illustrated with the fold change and *p*-value between the *cyp17a1*+/+ group and the *cyp17a1*−/− group (the fold change was calculated as the expression of the *cyp17a1*−/− group relative to that of the *cyp17a1*+/+ group). 

### 4.4. Validation of Differential Gene Expression by Quantitative Real-Time PCR

Total RNA was extracted from zebrafish fish using TRIzol reagent (15596-026; Invitrogen, Carlsbad, CA, USA) following a previously described standard protocol [12]. Each fish serves as an independent sample. We synthesized cDNA using One-Step gDNA Removal and cDNA Synthesis SuperMix (AE311-02; Transgen Biotech, Beijing, China). Quantitative real-time PCR was performed using SYBR Green Real-Time PCR Mix (AQ131-01; Transgen Biotech) and a real-time PCR system (Bio-Rad, Hercules, CA, USA). The housekeeping gene *β-actin* was used as endogenous control, and the expression level of target gene was calculated as the fold change relative to *β-actin* [46]. The primers used in this study are listed in Table 1.

### 4.5. 17β-Estradiol Administration

The fish were treated with 0.1 μg/L 17 β-estradiol (E8875, Sigma-Aldrich, St. Louis, MO, USA) from 16 to 23 dpf [12]. Briefly, 200 juvenile zebrafish at 16 dpf from all three genotypes (*cyp17a1*+/+, *cyp17a1*+/−, and *cyp17a1*−/−) were placed in a 10 L tank containing 0.1 μg/L 17 β-estradiol. At 23 dpf, images were captured by a Leica SP8 confocal microscope, and the caudal fin of the fish was cut for genotyping.

### 4.6. Estrogen Receptor Inhibitor Administration

The fish were treated with 500 ng/L tamoxifen (T137974, Aladdin, Shanghai, China) from 16 to 23 dpf according to a previous study [50]. Briefly, 200 juvenile offspring from the *Tg(piwil1:EGFP-nanos3UTR)* strain were placed in a 10 L tank containing 500 ng/L tamoxifen. At 23 dpf, images were captured by a Leica SP8 confocal microscope.

### 4.7. Statistical Analysis

Each experiment was performed in triplicate. When the comparison was conducted between *cyp17a1*+/+ fish and *cyp17a1*−/− fish, *cyp19a1a*+/+ fish and *cyp19a1a*−/− fish, or chemical reagent-untreated and -treated fish, the first mentioned fish of two was used as the control. All analyses were performed using the GraphPad Prism 6.0 software program. Statistical significance of differences was determined using two-tailed Student’s *t*-test for paired comparisons and one-way ANOVA, followed by Fisher’s LSD test for multiple comparisons. For all statistical comparisons, *p* < 0.05 indicates a significant difference. Significant differences marked with asterisks and letters were analyzed using Student’s *t*-test for paired comparisons and one-way ANOVA, followed by Fisher’s LSD test for multiple comparisons, respectively. Results are expressed as the mean ± standard error of the mean (SEM).

## Figures and Tables

**Table 1:** Primers used in this study.

Gene	Primer Direction ^a^ and Sequence (5′-3′)	Product Size (bp ^b^)	Reference
qPCR			
*a* *mh*	F: GCTGGGGAACTGGGGAAAAT	198	[47]
	R: CGGTAGGGAATGCTTTGGGA		
*g* *sdf*	F: GAACGCTCCTGAATCCACAGAC	210	[48]
	R: AATGACTCCCGCAGATGCTC		
*β-actin*	F: ACTCAGGATGCGGAAACTGG	118	[49]
	R: AGGGCAAAGTGGTAAACGCT		

^a^ F: forward; R: reverse. ^b^ base pair.

## Data Availability

The data underlying this article are available in the article and online Supplementary Material.

## Supplementary Materials

The supporting information can be downloaded at: https://www.mdpi.com/article/10.3390/ijms25031740/s1.

## References

[1] Dai S., Qi S., Wei X., Liu X., Li Y., Zhou X., Xiao H., Lu B., Wang D., Li M. (2021). Germline sexual fate is determined by the antagonistic action of *dmrt1* and *foxl3*/*foxl2* in tilapia. Development.

[2] Bell G. (1982). The Masterpiece of Nature: The Evolution and Genetics of Sexuality.

[3] Kikuchi K., Hamaguchi S. (2013). Novel sex-determining genes in fish and sex chromosome evolution. Dev. Dyn..

[4] Bull J.J. (1985). Sex determining mechanisms: An evolutionary perspective. Experientia.

[5] Liew W.C., Orban L. (2014). Zebrafish sex: A complicated affair. Brief. Funct. Genom..

[6] Vandeputte M., Dupont-Nivet M., Chavanne H., Chatain B. (2007). A polygenic hypothesis for sex determination in the European sea bass *Dicentrarchus labrax*. Genetics.

[7] Ser J.R., Roberts R.B., Kocher T.D. (2010). Multiple interacting loci control sex determination in lake Malawi cichlid fish. Evolution.

[8] Guerriero G. (2009). Vertebrate sex steroid receptors: Evolution, ligands, and neurodistribution. Ann. N. Y. Acad. Sci..

[9] Wilson C.A., High S.K., McCluskey B.M., Amores A., Yan Y.-l., Titus T.A., Anderson J.L., Batzel P., Carvan M.J., Schartl M. (2014). Wild sex in zebrafish: Loss of the natural sex determinant in domesticated strains. Genetics.

[10] Fenske M., Segner H. (2004). Aromatase modulation alters gonadal differentiation in developing zebrafish (*Danio rerio*). Aquat. Toxicol..

[11] Uchida D., Yamashita M., Kitano T., Iguchi T. (2004). An aromatase inhibitor or high water temperature induce oocyte apoptosis and depletion of P450 aromatase activity in the gonads of genetic female zebrafish during sex-reversal. Comp. Biochem. Physiol. A Mol. Integr. Physiol..

[12] Zhai G., Shu T., Xia Y., Lu Y., Shang G., Jin X., He J., Nie P., Yin Z. (2018). Characterization of Sexual Trait Development in cyp17a1-Deficient Zebrafish. Endocrinology.

[13] Lau E.S.-W., Zhang Z., Qin M., Ge W. (2016). Knockout of Zebrafish Ovarian Aromatase Gene (cyp19a1a) by TALEN and CRISPR/Cas9 Leads to All-male Offspring Due to Failed Ovarian Differentiation. Sci. Rep..

[14] Dranow D.B., Hu K., Bird A.M., Lawry S.T., Adams M.T., Sanchez A., Amatruda J.F., Draper B.W. (2016). Bmp15 Is an Oocyte-Produced Signal Required for Maintenance of the Adult Female Sexual Phenotype in Zebrafish. PLoS Genet..

[15] Yin Y., Tang H., Liu Y., Chen Y., Li G., Liu X., Lin H. (2017). Targeted Disruption of Aromatase Reveals Dual Functions of cyp19a1a During Sex Differentiation in Zebrafish. Endocrinology.

[16] Yu G., Zhang D., Liu W., Wang J., Liu X., Zhou C., Gui J., Xiao W. (2018). Zebrafish androgen receptor is required for spermatogenesis and maintenance of ovarian function. Oncotarget.

[17] Tang H., Chen Y., Wang L., Yin Y., Li G., Guo Y., Liu Y., Lin H., Cheng C.H.K., Liu X. (2018). Fertility impairment with defective spermatogenesis and steroidogenesis in male zebrafish lacking androgen receptor. Biol. Reprod..

[18] Webster K.A., Schach U., Ordaz A., Steinfeld J.S., Draper B.W., Siegfried K.R. (2017). Dmrt1 is necessary for male sexual development in zebrafish. Dev. Biol..

[19] Lin Q.H., Mei J., Li Z., Zhang X.M., Zhou L., Gui J.F. (2017). Distinct and Cooperative Roles of amh and dmrt1 in Self-Renewal and Differentiation of Male Germ Cells in Zebrafish. Genetics.

[20] Skaar K.S., Nobrega R.H., Magaraki A., Olsen L.C., Schulz R.W., Male R. (2011). Proteolytically activated, recombinant anti-mullerian hormone inhibits androgen secretion, proliferation, and differentiation of spermatogonia in adult zebrafish testis organ cultures. Endocrinology.

[21] Rodríguez-Marí A., Cañestro C., BreMiller R.A., Nguyen-Johnson A., Asakawa K., Kawakami K., Postlethwait J.H. (2010). Sex reversal in zebrafish *fancl* mutants is caused by Tp53-mediated germ cell apoptosis. PLoS Genet..

[22] Zhang Z.W., Zhu B., Chen W.T., Ge W. (2020). Anti-Mullerian hormone (Amh/amh) plays dual roles in maintaining gonadal homeostasis and gametogenesis in zebrafish. Mol. Cell. Endocrinol..

[23] Zhang Z.W., Wu K., Ren Z.Q., Ge W. (2020). Genetic evidence for Amh modulation of gonadotropin actions to control gonadal homeostasis and gametogenesis in zebrafish and its noncanonical signaling through Bmpr2a receptor. Development.

[24] Yan Y.L., Desvignes T., Bremiller R., Wilson C., Dillon D., High S., Draper B., Buck C.L., Postlethwait J. (2017). Gonadal soma controls ovarian follicle proliferation through Gsdf in zebrafish. Dev. Dynam..

[25] Clelland E., Peng C. (2009). Endocrine/paracrine control of zebrafish ovarian development. Mol. Cell. Endocrinol..

[26] Orban L., Sreenivasan R., Olsson P.E. (2009). Long and winding roads: Testis differentiation in zebrafish. Mol. Cell. Endocrinol..

[27] Kossack M.E., Draper B.W. (2019). Genetic regulation of sex determination and maintenance in zebrafish (Danio rerio). Curr. Top. Dev. Biol..

[28] Ye D., Zhu L., Zhang Q., Xiong F., Wang H., Wang X., He M., Zhu Z., Sun Y. (2019). Abundance of Early Embryonic Primordial Germ Cells Promotes Zebrafish Female Differentiation as Revealed by Lifetime Labeling of Germline. Mar. Biotechnol..

[29] Carlson R.W. (1997). Scientific review of tamoxifen. Overview from a medical oncologist. Semin. Oncol..

[30] Xu S., Xie F., Tian L., Fallah S., Babaei F., Manno S.H.C., Manno F.A.M., Zhu L., Wong K.F., Liang Y. (2020). Estrogen accelerates heart regeneration by promoting the inflammatory response in zebrafish. J. Endocrinol..

[31] Yin N., Jin X., He J., Yin Z. (2009). Effects of adrenergic agents on the expression of zebrafish (*Danio rerio*) vitellogenin Ao1. Toxicol. Appl. Pharmacol..

[32] Chen W., Liu L., Ge W. (2017). Expression analysis of growth differentiation factor 9 (*Gdf9*/*gdf9*), anti-mullerian hormone (Amh/amh) and aromatase (Cyp19a1a/cyp19a1a) during gonadal differentiation of the zebrafish, Danio rerio. Biol. Reprod..

[33] Uchida D., Yamashita M., Kitano T., Iguchi T. (2002). Oocyte apoptosis during the transition from ovary-like tissue to testes during sex differentiation of juvenile zebrafish. J. Exp. Biol..

[34] Dai X., Jin X., Chen X., He J., Yin Z. (2015). Sufficient numbers of early germ cells are essential for female sex development in zebrafish. PLoS ONE.

[35] Tzung K.W., Goto R., Saju J.M., Sreenivasan R., Saito T., Arai K., Yamaha E., Hossain M.S., Calvert M.E.K., Orban L. (2015). Early depletion of primordial germ cells in zebrafish promotes testis formation. Stem Cell Rep..

[36] Slanchev K., Stebler J., de la Cueva-Mendez G., Raz E. (2005). Development without germ cells: The role of the germ line in zebrafish sex differentiation. Proc. Natl. Acad. Sci. USA.

[37] Siegfried K.R., Nusslein-Volhard C. (2008). Germ line control of female sex determination in zebrafish. Dev. Biol..

[38] Ding Y., Yixuan T., Houpeng W., Mudan H., Yaqing W., Zhengfang C., Zhenxia C., Yonghua S. (2022). A landscape of differentiated biological processes involved in the initiation of sex differentiation in zebrafish. Water Biol. Secur..

[39] Brion F., Tyler C.R., Palazzi X., Laillet B., Porcher J.M., Garric J., Flammarion P. (2004). Impacts of 17beta-estradiol, including environmentally relevant concentrations, on reproduction after exposure during embryo-larval-, juvenile- and adult-life stages in zebrafish (*Danio rerio*). Aquat. Toxicol..

[40] Wu K., Song W.Y., Zhang Z.W., Ge W. (2020). Disruption of *dmrt1* rescues the all-male phenotype of the *cyp19a1a* mutant in zebrafish—A novel insight into the roles of aromatase/estrogens in gonadal differentiation and early folliculogenesis. Development.

[41] Wang Y., Ye D., Zhang F., Zhang R., Zhu J., Wang H., He M., Sun Y. (2022). *Cyp11a2* Is Essential for Oocyte Development and Spermatogonial Stem Cell Differentiation in Zebrafish. Endocrinology.

[42] Yan Y.-L., Titus T., Desvignes T., BreMiller R., Batzel P., Sydes J., Farnsworth D., Dillon D., Wegner J., Phillips J.B. (2021). A fish with no sex: Gonadal and adrenal functions partition between zebrafish NR5A1 co-orthologs. Genetics.

[43] Che H., Ma C., Li H., Yu F., Wei Y., Chen H., Wu J., Ren Y. (2022). Rebalance of the Polyamine Metabolism Suppresses Oxidative Stress and Delays Senescence in Nucleus Pulposus Cells. Oxidative Med. Cell. Longev..

[44] Nakajin S., Hall P.F., Onoda M. (1981). Testicular microsomal cytochrome P-450 for C21 steroid side chain cleavage. Spectral and binding studies. J. Biol. Chem..

[45] Westerfield M. (2020). The Zebrafish Book, a Guide for the Laboratory Use of Zebrafish (Danio rerio) OR.

[46] Livak K.J., Schmittgen T.D. (2001). Analysis of relative gene expression data using real-time quantitative PCR and the 2(-Delta Delta C(T)) Method. Methods.

[47] Zhai G., Shu T., Xia Y., Jin X., He J., Yin Z. (2017). Androgen signaling regulates the transcription of anti-Müllerian hormone via synergy with SRY-related protein SOX9A. Sci. Bull..

[48] Presslauer C., Nagasawa K., Dahle D., Babiak J., Fernandes J.M.O., Babiak I. (2014). Induced autoimmunity against gonadal proteins affects gonadal development in juvenile zebrafish. PLoS ONE.

[49] Shi C., Lu Y., Zhai G., Huang J., Shang G., Lou Q., Li D., Jin X., He J., Du Z. (2020). Hyperandrogenism in POMCa-deficient zebrafish enhances somatic growth without increasing adiposity. J. Mol. Cell Biol..

[50] Xia L., Zheng L., Zhou J.L. (2016). Transcriptional and morphological effects of tamoxifen on the early development of zebrafish (*Danio rerio*). J. Appl. Toxicol..

