# Supplementary material for: Estrogen Signaling Inhibits the Expression of anti-Müllerian hormone (amh) and gonadal-soma-derived factor (gsdf) during the Critical Time of Sexual Fate Determination in Zebrafish
Source: Int J Mol Sci. 2024 Feb 1;25(3):1740. doi: 10.3390/ijms25031740 (PMC10855942; doi:10.3390/ijms25031740)
Supplement: Supplementary file 1 [file ijms-25-01740-s001.zip › ijms-2790757-supplementary.pdf]

Supplemental Files

Supplemental Table

Supplemental Table S1 The differentially expressed genes in *cyp17a1*+/+ fish and *cyp17a1*-/- fish of PGC-rich group

Gene Name	log2FoldChange	P-Value	Description
si:dkey-169i5.4	6.0219	0.0000	si:dkey-169i5.4
AL831726.2	5.8342	0.0000	uncharacterized LOC101882696
zmp:0000000845	5.5546	0.0000	zmp:0000000845
pdgfaa	5.2831	0.0000	platelet-derived growth factor alpha polypeptide a
si:ch211-156p11.1	5.0465	0.0028	si:ch211-156p11.1
CR394546.4	5.0317	0.0029	GTPase IMAP family member 4-like
MFAP4 (1 of many)	4.8668	0.0000	si:zfos-2330d3.1
BX324230.2	4.8600	0.0000	-
si:dkeyp-46h3.5	4.7386	0.0031	si:dkeyp-46h3.5
si:ch211-197g15.6	4.5677	0.0252	si:ch211-197g15.6
si:ch73-236c18.6	4.3639	0.0000	si:ch73-236c18.6
ccl39.5	4.2334	0.0411	chemokine (C-C motif) ligand 39, duplicate 5
CABZ01009512.2	4.1475	0.0072	NACHT, LRR and PYD domains-containing protein 3-like
BX248128.2	4.0331	0.0373	-
si:dkey-88j15.3	3.9833	0.0012	si:dkey-88j15.3
si:ch211-226h7.5	3.7529	0.0001	si:ch211-226h7.5
gpr84	3.7298	0.0120	G protein-coupled receptor 84
klhl38b	3.6462	0.0004	kelch-like family member 38b
si:ch211-270n8.1	3.6345	0.0007	si:ch211-270n8.1

CR293501.2	3.5808	0.0218	-
capn2l	3.5237	0.0000	calpain 2, (m/II) large subunit, like
wnt3a	3.4655	0.0483	wingless-type MMTV integration site family, member3A
si:dkey-56m15.8	3.4135	0.0025	si:dkey-56m15.8
zmp:0000000650	3.3001	0.0188	zmp:0000000650
CU571328.1	3.1586	0.0378	heterogeneous nuclear ribonucleoprotein A1-like
zgc:152753	3.1072	0.0249	zgc:152753
klhl10a	3.0354	0.0033	kelch-like family member 10a
si:ch73-106k19.5	3.0096	0.0001	si:ch73-106k19.5
CR749163.1	2.9387	0.0012	uncharacterized LOC108179108
slco1f3	2.9194	0.0021	solute carrier organic anion transporter family,member 1F3
BX908782.1	2.9102	0.0314	CD59 glycoprotein-like
si:ch211-66i15.5	2.7973	0.0258	si:ch211-66i15.5
si:ch73-364h19.2	2.7313	0.0006	si:ch73-364h19.2
BX248410.1	2.7227	0.0198	Schwann cell myelin protein-like
BX321875.3	2.6792	0.0010	-
si:dkeyp-46h3.3	2.5864	0.0219	si:dkeyp-46h3.3
si:ch211-227e10.3	2.5827	0.0017	si:ch211-227e10.3
si:ch211-226h7.8	2.5742	0.0069	si:ch211-226h7.8
BX324216.3	2.5110	0.0332	si:ch211-222k6.1
si:ch211-236p5.3	2.5004	0.0027	si:ch211-236p5.3
si:ch73-367f21.6	2.4556	0.0375	si:ch73-367f21.6
si:dkey-262g12.7	2.4193	0.0289	si:dkey-262g12.7
si:dkey-207m2.4	2.4050	0.0288	si:dkey-207m2.4
cyp19a1a	2.3989	0.0023	cytochrome P450, family 19, subfamily A,polypeptide 1a

si:ch211-227e10.1	2.3863	0.0052	si:ch211-227e10.1
si:dkey-39a18.1	2.3424	0.0243	si:dkey-39a18.1
si:dkey-217f16.1	2.3276	0.0027	si:dkey-217f16.1
iqch	2.3243	0.0010	IQ motif containing H
si:dkey-7c18.24	2.3121	0.0001	si:dkey-7c18.24
zgc:136605	2.2951	0.0473	zgc:136605
si:ch211-212k5.1	2.2782	0.0009	si:ch211-212k5.1
BX005442.3	2.2683	0.0417	-
CABZ01033178.1	2.2579	0.0014	-
si:dkey-286j15.1	2.2541	0.0059	si:dkey-286j15.1
zmp:0000001020	2.2363	0.0045	zmp:0000001020
ebf3b	2.2319	0.0271	early B cell factor 3b
ddx41	2.2068	0.0180	DEAD (Asp-Glu-Ala-Asp) box polypeptide 41
si:ch73-236c18.7	2.1869	0.0089	si:ch73-236c18.7
cfh	2.1362	0.0041	complement factor H
arg1	2.1287	0.0493	arginase 1
irgf3	2.1246	0.0406	immunity-related GTPase family, f3
faslg	2.1213	0.0334	Fas ligand (TNF superfamily, member 6)
amh	2.1162	0.0003	anti-Mullerian hormone
CABZ01074397.1	2.0510	0.0228	NACHT, LRR and PYD domains-containing protein 3-like
syt16	2.0482	0.0427	synaptotagmin XVI
c1r	2.0452	0.0025	complement component 1, r subcomponent
BX323596.1	2.0369	0.0100	C-X-C motif chemokine 11-6-like
si:dkey-88n24.6	2.0135	0.0240	si:dkey-88n24.6
si:ch211-181d7.1	2.0008	0.0092	si:ch211-181d7.1
si:dkey-16p6.1	1.9861	0.0349	si:dkey-16p6.1
zgc:174314	1.9753	0.0004	zgc:174314

si:dkey-56m15.9	1.9692	0.0137	si:dkey-56m15.9
cbln7	1.9377	0.0018	cerebellin 7
AL935186.1	1.9163	0.0321	-
pdk3b	1.9053	0.0035	pyruvate dehydrogenase kinase, isozyme 3b
si:ch1073-358c10.1	1.9002	0.0037	si:ch1073-358c10.1
gja8a	1.8985	0.0134	gap junction protein alpha 8 paralog a
ssh1a	1.8804	0.0241	slingshot protein phosphatase 1a
si:dkey-172k15.6	1.8704	0.0086	si:dkey-172k15.6
tnfaip2b	1.8611	0.0162	tumor necrosis factor, alpha-induced protein 2b
si:ch211-226h7.3	1.8448	0.0339	si:ch211-226h7.3
si:dkey-238k10.1	1.8270	0.0236	si:dkey-238k10.1
BX005442.2	1.8180	0.0136	-
slc2a6	1.8119	0.0116	solute carrier family 2 (facilitated glucosetransporter), member 6
zgc:171679	1.8041	0.0000	zgc:171679
si:ch211-181d7.3	1.7794	0.0069	si:ch211-181d7.3
cfhl1	1.7671	0.0060	complement factor H like 1
si:ch211-223g7.2	1.7650	0.0137	si:ch211-223g7.2
MFAP4 (1 of many)	1.7615	0.0270	si:ch1073-110a20.2
btr12	1.7613	0.0116	bloodthirsty-related gene family, member 12
ptprc	1.7441	0.0000	protein tyrosine phosphatase, receptor type, C
eve1	1.7413	0.0287	even-skipped-like1
epm2a	1.7379	0.0061	epilepsy, progressive myoclonus type 2A, Laforadisease (laforin)
ifit9	1.7372	0.0340	interferon-induced protein with tetratricopeptiderepeats 9

ly6m6	1.7244	0.0001	lymphocyte antigen 6 family member M6
zgc:165583	1.7161	0.0234	zgc:165583
si:dkey-9i23.16	1.7099	0.0205	si:dkey-9i23.16
cfhl2	1.7085	0.0002	complement factor H like 2
gabrr2b	1.7037	0.0112	gamma-aminobutyric acid (GABA) A receptor, rho 2b
rhoj	1.6941	0.0436	ras homolog family member J
si:dkeyp-75b4.10	1.6756	0.0010	si:dkeyp-75b4.10
si:dkey-13p1.3	1.6642	0.0389	si:dkey-13p1.3
gja3	1.6639	0.0292	gap junction protein, alpha 3
si:dkey-23c22.5	1.6475	0.0350	si:dkey-23c22.5
capn8	1.6389	0.0028	calpain 8
si:ch73-236c18.5	1.6361	0.0081	si:ch73-236c18.5
micall1b.2	1.6184	0.0010	MICAL like 1b, duplicate 2
acot19	1.6057	0.0000	acyl-CoA thioesterase 19
CABZ01033206.1	-1.5034	0.0007	V-set and transmembrane domain containing 5
pde9al	-1.5053	0.0312	phosphodiesterase 9A like
si:dkey-33c14.6	-1.5405	0.0437	si:dkey-33c14.6
si:ch211-227e10.6	-1.5662	0.0476	si:ch211-227e10.6
si:ch73-359m17.6	-1.5679	0.0000	si:ch73-359m17.6
cyp46a1.4	-1.5707	0.0132	cytochrome P450, family 46, subfamily A,polypeptide 1, tandem duplicate 4
si:dkey-16p6.1	-1.5717	0.0187	si:dkey-16p6.1
slx4ip	-1.6027	0.0000	SLX4 interacting protein
si:ch211-133n4.9	-1.6095	0.0218	si:ch211-133n4.9
mchr1b	-1.6131	0.0360	melanin-concentrating hormone receptor 1b
si:dkey-12e7.4	-1.6361	0.0027	si:dkey-12e7.4
si:dkey-7i4.15	-1.6367	0.0065	si:dkey-7i4.15

si:ch211-191a16.5	-1.6482	0.0291	si:ch211-191a16.5
si:ch211-214b16.2	-1.6769	0.0054	si:ch211-214b16.2
si:ch211-281l24.3	-1.6949	0.0071	si:ch211-281l24.3
zgc:162184	-1.7186	0.0000	zgc:162184
gp1bb	-1.7341	0.0002	glycoprotein Ib platelet subunit beta
kcnj13	-1.7489	0.0181	potassium inwardly-rectifying channel, subfamily J,member 13
sparcl2	-1.8181	0.0387	SPARC-like 2
hpdl	-1.8189	0.0016	4-hydroxyphenylpyruvate dioxygenase-like
im:6904045	-1.8229	0.0399	im:6904045
si:zfos-364h11.2	-1.8353	0.0257	si:zfos-364h11.2
si:ch211-125e6.8	-1.8566	0.0127	si:ch211-125e6.8
cyp2k8	-1.8841	0.0061	cytochrome P450, family 2, subfamily K, polypeptide8
ypel5	-1.9375	0.0000	yippee-like 5
si:dkey-121n8.7	-1.9476	0.0158	si:dkey-121n8.7
CU855552.1	-1.9706	0.0125	-
hyal2a	-1.9784	0.0334	hyaluronidase 2a
scamp2l	-2.0288	0.0000	secretory carrier membrane protein 2, like
csrp3	-2.0535	0.0434	cysteine and glycine-rich protein 3 (cardiac LIMprotein)
si:dkey-202e17.1	-2.0548	0.0000	si:dkey-202e17.1
si:dkey-43p13.5	-2.0770	0.0385	si:dkey-43p13.5
si:ch211-182p11.1	-2.1290	0.0000	si:ch211-182p11.1
si:ch211-187g4.1	-2.1542	0.0435	si:ch211-187g4.1
nlrc8	-2.1715	0.0070	NLR family CARD domain containing 8
si:ch211-202m22.1	-2.1928	0.0331	si:ch211-202m22.1
si:ch211-208f21.3	-2.1947	0.0013	si:ch211-208f21.3
si:ch73-181m17.1	-2.2842	0.0154	si:ch73-181m17.1

zgc:173709	-2.3769	0.0022	zgc:173709
znf1026	-2.5295	0.0096	zinc finger protein 1026
si:ch73-329n5.1	-2.5680	0.0106	si:ch73-329n5.1
si:ch73-174h16.5	-2.5688	0.0234	si:ch73-174h16.5
cyp2k18	-2.5745	0.0415	cytochrome P450, family 2, subfamily K, polypeptide18
si:dkeyp-51b9.3	-2.7990	0.0000	si:dkeyp-51b9.3
ddx43	-2.8361	0.0220	DEAD (Asp-Glu-Ala-Asp) box polypeptide 43
fam221a	-2.8866	0.0224	family with sequence similarity 221, member A
ftr90	-2.9192	0.0000	finTRIM family, member 90
FQ311879.1	-2.9648	0.0328	-
zgc:173837	-3.2774	0.0152	zgc:173837
PAOX	-3.3767	0.0135	si:dkey-275b16.2
znf1066	-3.5445	0.0341	zinc finger protein 1066
nlrc9	-3.5715	0.0003	NLR family CARD domain containing 9
zgc:172053	-3.6509	0.0124	zgc:172053
BX649448.3	-3.6923	0.0001	-
si:dkey-11o15.8	-3.8152	0.0367	si:dkey-11o15.8
si:dkey-105i14.1	-4.0346	0.0000	si:dkey-105i14.1
zbtb7c	-4.1942	0.0433	zinc finger and BTB domain containing 7C
CR792441.1	-4.2796	0.0006	-
znf1109	-4.2827	0.0340	zinc finger protein 1109
znf1068	-4.6515	0.0106	zinc finger protein 1068
si:ch211-108d22.2	-6.0086	0.0001	si:ch211-108d22.2
zgc:195170	-6.5129	0.0028	zgc:195170
taco1	-7.2600	0.0012	translational activator of mitochondrially encodedcytochrome c oxidase I

si:ch211-197e7.1	-7.3674	0.0000	si:ch211-197e7.1

Supplemental Table S2 The differentially expressed genes in *cyp17a1*+/+ fish and *cyp17a1*-/- fish of PGC-less group

Gene Name	log2FoldChange	P-Value	Description
steap4	1.8523	0.0003	STEAP family member 4
rrm2	6.0181	0.0003	ribonucleotide reductase M2 polypeptide
dap1b	1.8067	0.0008	death associated protein 1b
nlrc8	6.5234	0.0015	NLR family CARD domain containing 8
jac9	2.8205	0.0021	jacalin 9
mettl8	1.9152	0.0036	methyltransferase like 8
frmd7	4.2683	0.0070	FERM domain containing 7
si:dkey-16p6.1	2.2940	0.0076	si:dkey-16p6.1
iqcc	1.7771	0.0078	IQ motif containing C
si:ch211-167j9.4	4.8078	0.0081	si:ch211-167j9.4
si:ch211-281l24.3	4.2091	0.0082	si:ch211-281l24.3
pimr70	4.9566	0.0106	Pim proto-oncogene, serine/threonine kinase,related 70
si:dkey-11o1.6	3.3689	0.0112	si:dkey-11o1.6
MPP4 (1 of many)	1.8669	0.0112	si:ch211-222n4.6
drc1	2.6109	0.0139	dynein regulatory complex subunit 1 homolog(Chlamydomonas)
igflr1	1.6604	0.0153	IGF-like family receptor 1
si:ch73-364h19.2	2.7328	0.0157	si:ch73-364h19.2
itgae.1	2.8743	0.0167	integrin, alpha E, tandem duplicate 1
zgc:112332	1.6483	0.0223	zgc:112332
hbbe1.2	3.4233	0.0237	hemoglobin beta embryonic-1.2
si:ch73-334d15.4	3.5051	0.0246	si:ch73-334d15.4
si:dkey-54j5.2	4.5916	0.0248	si:dkey-54j5.2
si:ch211-51h4.2	3.1411	0.0286	si:ch211-51h4.2

stbd1	1.6653	0.0295	starch binding domain 1
esr2a	1.5116	0.0298	estrogen receptor 2a
gbe1a	1.7730	0.0299	glucan (1,4-alpha-), branching enzyme 1a
dcst1	4.7709	0.0302	DC-STAMP domain containing 1
itgb8	1.6599	0.0311	integrin, beta 8
LO018340.1	1.5437	0.0325	ADAMTS-like 4
znf1040	2.4164	0.0347	zinc finger protein 1040
si:ch211-106h4.5	1.9188	0.0358	si:ch211-106h4.5
si:dkey-188i13.6	1.7934	0.0371	si:dkey-188i13.6
odf3b	2.6661	0.0377	outer dense fiber of sperm tails 3B
wisp1b	1.8297	0.0397	WNT1 inducible signaling pathway protein 1b
unm_sa821	2.3211	0.0400	un-named sa821
loxl3a	2.0446	0.0402	lysyl oxidase-like 3a
agbl4	2.0998	0.0412	ATP/GTP binding protein-like 4
BX284696.1	4.4816	0.0455	-
her3	4.4340	0.0461	hairy-related 3
gaa	1.5895	0.0463	glucosidase, alpha; acid (Pompe disease, glycogenstorage disease type II)
zgc:112966	1.9899	0.0466	zgc:112966
tm4sf18	1.6486	0.0473	transmembrane 4 L six family member 18
ggact.2	-8.8094	0.0000	gamma-glutamylamine cyclotransferase, tandemduplicate 2
zgc:153169	-2.3733	0.0000	zgc:153169
fthl28	-1.8664	0.0000	ferritin, heavy polypeptide-like 28
pm20d1.1	-1.7654	0.0000	peptidase M20 domain containing 1, tandem duplicate1
zgc:172053	-2.1320	0.0002	zgc:172053
si:ch211-181d7.1	-2.0789	0.0002	si:ch211-181d7.1

ctsba	-1.6369	0.0003	cathepsin Ba
CABZ01009512.1	-2.0616	0.0003	ribonuclease inhibitor-like
si:ch211-193k19.2	-5.8071	0.0005	si:ch211-193k19.2
si:ch211-213a13.5	-3.7710	0.0006	si:ch211-213a13.5
si:ch211-196c10.15	-3.5365	0.0006	si:ch211-196c10.15
rad21l1	-2.9016	0.0014	RAD21 cohesin complex component like 1
si:ch211-11p18.6	-5.3704	0.0024	si:ch211-11p18.6
zte38	-5.0861	0.0030	zebrafish testis-expressed 38
pdgfaa	-4.5812	0.0031	platelet-derived growth factor alpha polypeptide a
si:ch211-214b16.3	-1.6244	0.0032	si:ch211-214b16.3
galm	-1.5123	0.0034	galactose mutarotase
hoxb13a	-1.6627	0.0036	homeobox B13a
si:ch211-217k17.12	-2.6015	0.0038	si:ch211-217k17.12
asb14b	-1.5255	0.0040	ankyrin repeat and SOCS box containing 14b
si:dkey-19a16.4	-1.9286	0.0056	si:dkey-19a16.4
znfl1l	-4.2590	0.0084	zinc finger-like gene 1l
si:dkey-28g23.6	-2.2315	0.0097	si:dkey-28g23.6
CR848788.1	-3.3843	0.0102	-
si:ch211-250k18.7	-4.1613	0.0119	si:ch211-250k18.7
jac1	-4.4682	0.0122	jacalin 1
CELA1 (1 of many)	-2.8395	0.0128	zgc:112302
prf1.9	-2.6426	0.0132	perforin 1.9
pbld2	-1.5794	0.0134	phenazine biosynthesis-like protein domaincontaining 2
si:ch211-160j14.2	-2.1284	0.0139	si:ch211-160j14.2
ddx43	-3.6161	0.0142	DEAD (Asp-Glu-Ala-Asp) box polypeptide 43

si:ch211-76m11.8	-1.6083	0.0142	si:ch211-76m11.8
PAOX	-2.3037	0.0153	si:dkey-275b16.2
si:ch211-66i15.5	-3.5124	0.0169	si:ch211-66i15.5
BX005256.1	-4.8441	0.0182	phospholipase A2 inhibitor CNF-like
alox5b.2	-6.6833	0.0182	arachidonate 5-lipoxygenase b, tandem duplicate 2
MAP3K11	-1.7747	0.0232	si:cabz01078036.1
FO704779.1	-5.5552	0.0234	-
lrrc51	-3.3908	0.0247	leucine rich repeat containing 51
irf3	-1.5419	0.0248	interferon regulatory factor 3
ppp1r27b	-2.7039	0.0259	protein phosphatase 1, regulatory subunit 27b
fam110a	-1.6143	0.0263	family with sequence similarity 110, member A
caspbl	-1.6980	0.0290	caspase b, like
CU467905.1	-1.6061	0.0292	caspase-3-like
si:ch73-44m9.2	-2.4871	0.0302	si:ch73-44m9.2
si:ch211-181d7.3	-1.5325	0.0307	si:ch211-181d7.3
gstk4	-2.1989	0.0308	glutathione S-transferase kappa 4
crp6	-3.5040	0.0354	C-reactive protein 6
si:dkey-16p19.5	-2.8914	0.0356	si:dkey-16p19.5
si:dkey-193b15.5	-2.3566	0.0357	si:dkey-193b15.5
si:dkey-28d5.5	-1.6896	0.0366	si:dkey-28d5.5
ftr55	-2.0021	0.0378	finTRIM family, member 55
qdprb2	-4.0726	0.0382	quinoid dihydropteridine reductase b2
si:ch211-241n15.3	-2.8055	0.0387	si:ch211-241n15.3
tmigd1	-1.5798	0.0426	transmembrane and immunoglobulin domain containing1
zgc:173443	-5.4533	0.0432	zgc:173443

fam221a	-2.4132	0.0442	family with sequence similarity 221, member A
sycp2	-2.2203	0.0454	synaptonemal complex protein 2
zgc:153760	-2.2131	0.0454	zgc:153760
ripk3	-2.1700	0.0456	receptor-interacting serine-threonine kinase 3
si:dkey-78l4.2	-4.3316	0.0466	si:dkey-78l4.2
tbpl2	-3.3590	0.0470	TATA box binding protein like 2
AL928685.4	-3.3816	0.0475	-
grb7	-1.9300	0.0492	growth factor receptor bound protein 7
